# Medical Cannabis: Toward a New Policy and Health Model for an Ancient Medicine

**DOI:** 10.3389/fpubh.2022.904291

**Published:** 2022-05-27

**Authors:** Davide Fortin, Fabienne Marcellin, Patrizia Carrieri, Julien Mancini, Tangui Barré

**Affiliations:** ^1^University Paris 1 Sorbonne, Paris, France; ^2^Aix Marseille Univ, INSERM, IRD, SESSTIM, Sciences Economiques & Sociales de la Santé & Traitement de l'Information Médicale, ISSPAM, Marseille, France

**Keywords:** cannabis, drug policy, cannabis flowers, marijuana, real-word evidence, France, patient-reported outcomes (PROs)

## Introduction

Cannabis has been grown and exploited by mankind for its therapeutic properties since ancient times (1). Although a growing number of countries have approved cannabis-based products for medical use, high-quality evidence for cannabis itself (understood in this article as the unprocessed flowering tops of the plant) in this context is lacking, and only a few jurisdictions to date have approved the medical use of cannabis, mostly as magistral preparations (2). The reason for this lies in the large variation in cannabis material as a plant (3, 4). Real-word data on the medical use of cannabis could be of benefit to patients worldwide, healthcare professionals, policymakers, and researchers (3, 5, 6). In this article, we discuss these points and propose that the collection of patient-reported outcomes (PROs) could be a cornerstone of a medical cannabis policy.

## Cannabis, a Square PEG in the Round Hole of Evidence-Based Medicine that Relies Exclusively on Randomized Controlled Trials

The recent discoveries of cannabinoids (compounds interacting with cannabinoid receptors) and of the endocannabinoid system (ECS) have boosted the expansion of cannabis-related research (7). The ECS is a signaling network composed of cannabinoid receptors (CB1 and CB2), their ligands (endocannabinoids), and ligand synthesizing and degrading enzymes. It is a complex system, expressed through most organs, and is involved in many physiological pathways. Phytocannabinoids (cannabinoids from plants, generally from cannabis) interact with the ECS and may bring consequent health effects (8).

The two most abundant and studied phytocannabinoids are Δ9-tetrahydrocannabinol (THC) and cannabidiol (CBD). Evidence-based medicine (EBM) focusing on these purified “major” cannabinoids has led to the development of several drugs including dronabinol (THC), nabilone (synthetic analog of THC), Epidiolex (CBD), and nabiximols (a balanced mixture of THC and CBD). These drugs have been approved for a very limited number of conditions such as resistant epilepsy.

The therapeutic potential of cannabis is not limited to the effects of THC and CBD, or to their interactions with CB1 and CB2 (8). Numerous “minor” phytocannabinoids, such as Δ9-tetrahydrocannabivarin, cannabichromene, cannabigerol, and cannabinol, act on the ECS in ways which likely engender health effects (8, 9). However, far fewer studies have investigated their potential benefits. Phytocannabinoids may also interact with receptors other than cannabinoid receptors (8), and between-phytocannabinoid interactions have also been highlighted (10, 11).

In addition to phytocannabinoids, the *Cannabis* genus also produces non-cannabinoid compounds, including terpenoids (12), which may also be medically useful (13–15). The pharmacological contributions of minor cannabinoids and non-cannabinoid compounds have been highlighted and popularized under the term “entourage effect” (16, 17). Through synergistic mechanisms between different cannabis chemical components [over 500 have been identified to date (18, 19)], “full-spectrum” cannabis extracts may have different and potentially superior effects to those observed with purified major cannabinoids (16, 17).

Unlike purified cannabinoids, cannabis constitutes a mixture of multi-target active compounds that interact with each other. There is great genetic variability between cannabis plants (20). Each chemical variety of cannabis (chemovar or chemotype) has a specific profile of various cannabinoid and non-cannabinoid compounds depending on its genetic make-up (16, 21). This profile can change depending on pre- and post-harvest environmental factors (22–25). Accordingly, each of these various “cocktails” is likely to have a different impact (including adverse effects) on different people, according to the individual's genetic factors (26–28). This diversity in effects may match the diversity of individual needs, may help reduce treatment gaps, and may lessen the burden of therapeutic deadlocks.

Indications for treatment by cannabinoids, supported by low to moderate certainty of evidence, include chronic pain, some treatment resistant epilepsies, and nausea and vomiting caused by chemotherapy (29, 30). However, evidence is lacking for cannabis, for several reasons. First, in order to follow EBM principles, randomized-controlled trials (RCT) are needed. Very few RCT have been conducted with cannabis (31). RCT necessitates a stable, standardized, and characterized product to test against another product in a characterized sample of patients. However, standardization/characterization is a problem when dealing with plant material (32). Second, given the variability of cannabis materials, the external validity of RCT results would be highly questionable. Moreover, cannabis material available to researchers may be different to what is available to users (33). Third, RCT are expensive, and the lack of patentability for findings means little economic incentive to conduct such research (4). Fourth, there would be difficulties in interpreting the evidence because of the variety of compounds involved. Consequently, cannabis as a “regular” medicinal product (i.e., a drug delivered through prescription for a given condition and with a given posology) is unlikely to be approved in the foreseeable future.

## Real-Word is The New Evidence

Despite the lack of RCT-based evidence, many patient reports and large scale observational studies have attested to the medical effectiveness of cannabis (3, 34). We believe that in the context of medical cannabis, the current approach to EBM which sees RCT as the exclusive means for valid evidence of treatment effectiveness, needs to be reconsidered. More specifically, in the context of medical cannabis, we agree with the view that RCT are “intensive, expensive interventions delivered in leading medical centers by world-class experts and requiring very skilled intervention delivery and high fidelity, administered to uncomplicated, highly motivated patients, [which] cannot be expected to work equally well in the messy, real-world, under-resourced public health settings around the world dealing with complex comorbid patients living in stressful, non-supportive environments” (35).

Real-world evidence, including PROs [i.e., where health status is reported directly by the patient, without interpretation by a clinician or anyone else (36)], is now building up to a pattern of evidence, emphasizing the effectiveness of using medical cannabis to treat pain syndromes as well as various psychiatric conditions (3). Real-world evidence can be used to complement RCT or to serve as a precursor to them in order to increase the speed at which evidence is generated, and to reduce costs (5). For instance, emulating randomized trials from large observational databases and statistical methods to account for bias (37) hold great promise for assessing cannabis' effectiveness (38, 39).

## Current Models of Regulatory Frameworks for Medical Cannabis

Currently, two main types of regulatory frameworks exist for medical cannabis: the accommodative North American framework, and the restrictive European one. In the U.S., citizen-initiated referenda have led to the legalization of medical cannabis, whereby therapy is dispensed according to state-level regulation (4). Initially permitted for a small number of health conditions, the list has progressively grown to the point where almost all adults can now access it where it is legal (40). Home cultivation, sometimes subject to quantity restriction and/or registration, is also permitted in some U.S. states. Currently, over 50% of U.S. states have fully authorized the medical use of cannabis (2). In Canada, the medical cannabis market was designed by policymakers, but similarly to the U.S., it allows patients to buy cannabis from a licensed producer. Medical practitioners and registered nurses are responsible for providing a document that allows access. The major differences with the U.S. are the need for a bona fide doctor-patient relationship, and the lack of retail distribution, which can only be home delivered (41). Patients in Canada can also register to produce a limited amount of cannabis for their own medical purposes, or designate someone to produce it on their behalf.

On the contrary, in European countries where medical cannabis (or cannabis-based products) is legal, there are significant restrictions both on eligible medical conditions and on the types of products available (40). Medical cannabis-based products are mostly made available through special access schemes and as a last intention treatment, meaning that the patient must have previously tried other commonly used treatment options. The most common authorized medical cannabis-based products are standardized drugs containing cannabinoids. Only six European countries (Czech Republic, Denmark, Italy, Netherlands, Portugal, and Germany) have established programs allowing patients to access cannabis (i.e., herbal preparations) (42). Italy and Netherlands only permit access to cannabis decontaminated through gamma-irradiation [and therefore undergoing a few changes in the terpene profile (43)] (4). Pharmaceutical products containing cannabinoids are usually reimbursed from the health system under specific conditions (44). Costs for cannabis can be reimbursed if conventional treatments have failed and under specific conditions (e.g., upon prior approval in Germany). In all the above-cited North American and European jurisdictions, most regulators allow physicians to decide which indications they will prescribe cannabis for (2).

## Toward Reciprocity: Where Practice Fuels Knowledge and Vice-Versa

Given the huge numbers of patients looking for symptom relief from different health conditions, the limitations of the European (access too restrictive) and North American [high risk of cannabis use disorder (45)] medical cannabis policies, need to be tackled (4). To ensure optimal use of medical cannabis and to best meet the needs of patients (e.g., symptom alleviation), healthcare professionals (e.g., providing a clear picture of cannabis' effects and indications for medical use), and society (e.g., potentially decreasing health-care reimbursement costs), it is essential to implement high-quality, structured and systematic collection of real-world evidence, especially PROs. Indeed, as PROs come directly from the patient, their evolution is likely to reflect users' satisfaction derived from a treatment, and its impact on quality of life. Health-related quality of life measures can then be analyzed and translated in terms of cost-effectiveness (or cost-utility), and contribute in guiding decision-makers (46–48). PROs measure outcomes important to patients than cannot be captured through clinical measures, and offer opportunities to ensure that the patients' voice is at the heart of the health-care model. Symptom (e.g., pain) alleviation is a common motive for cannabis use (rather than disease curation), and PROs are particularly fitted to assess severity of symptoms and/or associated distress (49–51). PROs can be collected through user-friendly self-administered questionnaires, including electronic ones (49, 50), and at home (52, 53). Therefore, collection of PROs may need virtually no training for patients and health professionals.

Efforts should be made to ensure collaboration between stakeholders, to establish a standard set of tools, measures and methods, to formulate a clear governance process for generating real-world evidence based on PROs, to minimize workload and technical complexity, to provide guidance on how to interpret and use data, and to ensure that patients and clinicians gain value from assessment through real-time access to PROs data so that treatment can be individually tailored (6). Collecting data on the cannabis chemovar used, and patients' patterns of use (i.e., route and frequency of administration) is also indispensable.

Proposing a complete model for medical cannabis (or full-spectrum extracts) care is out of the scope of the present article. Nevertheless, we suggest a few points to consider when creating or adapting medical cannabis policy. Given its use since antiquity, the safety profile of cannabis is well-known. However, while THC-related harms (54, 55) and the very real risk of dependence (45, 56) have been described in detail, drug-drug interactions remain under-documented (57). Accordingly, the individual benefit/risk ratio should precede any prescription through an assessment of potential contraindications or limitations. Minimization of combustion-based routes of administration should be emphasized (58, 59) and supported by providing appropriate material such as vaporizers (60).

We also suggest that incentives be implemented to combine the collection of PROs with the dispensing of medical cannabis, through a post-marketing type assessment. By doing so, an accessible, standardized, high-quality corpus of real-world evidence on the medical use of cannabis would be generated and grow with time and experience. By systematically collecting and documenting cases that were previously anecdotal, as well as by characterizing optimal patterns of use for conditions concerning large number of cases, such systematic data would inform and benefit both patients and physicians. We can therefore expect a high level of social acceptability of combining collection of PROs with the dispensing of medical cannabis, more or less in line with acceptability of participatory and/or community research frameworks.

## Final Considerations

In this opinion article, we discussed the difficult marrying the highly variable, multiple-component, and multiple-target drug that is cannabis to EBM which is currently exclusively based on RCT. In our opinion, the traditional empirical use of cannabis needs to be reconciled with an EBM which does not solely rely on RCT. We hypothesize that incorporating the collection of PROs into medical cannabis policy would benefit patients, healthcare professionals, and society.

## Author Contributions

DF, FM, PC, and JM designed the article and reviewed it. TB designed the article, wrote the manuscript draft, and reviewed it. All authors contributed to the article and approved the submitted version.

## Conflict of Interest

The authors declare that the research was conducted in the absence of any commercial or financial relationships that could be construed as a potential conflict of interest.

## Publisher's Note

All claims expressed in this article are solely those of the authors and do not necessarily represent those of their affiliated organizations, or those of the publisher, the editors and the reviewers. Any product that may be evaluated in this article, or claim that may be made by its manufacturer, is not guaranteed or endorsed by the publisher.
